# Stroke Mortality Attributable to Ambient Particulate Matter Pollution from 1990 to 2015 in China: An Age-Period-Cohort and Spatial Autocorrelation Analysis

**DOI:** 10.3390/ijerph14070772

**Published:** 2017-07-13

**Authors:** Lisha Luo, Junfeng Jiang, Ganshen Zhang, Lu Wang, Zhenkun Wang, Jin Yang, Chuanhua Yu

**Affiliations:** 1 Department of Epidemiology and Biostatistics, School of Health Sciences, Wuhan University, #185 Donghu Road, Wuhan 430071, China; 13006362970@163.com (L.L.); jiang0111@whu.edu.cn (J.J.); gszhang1991@163.com (G.Z.); ffdw_03@163.com (L.W.); wongzhenkun@gmail.com (Z.W.); jinan0218@163.com (J.Y.); 2 Global Health Institute, Wuhan University, #8 Donghu Road, Wuhan 430072, China

**Keywords:** stroke, PM_2.5_, mortality, age-period-cohort analysis, spatial autocorrelation

## Abstract

In this study, we analyzed the temporal and spatial variations of stroke mortality attributable to ambient particulate matter pollution (stroke mortality-PM_2.5_) in China from 1990 to 2015. Data were collected from the Global Burden of Disease (GBD) 2015 study and analyzed by an age-period-cohort model (APC) with an intrinsic estimator (IE) algorithm, as well as spatial autocorrelation based on the Geographic Information System. Based on APC analysis with the IE method, stroke mortality-PM_2.5_ increased exponentially with age, its relative risk reaching 42.85 (95% CI: 28.79, 63.43) in the 75–79 age group. The period effects showed a reversed V-shape and its highest relative risk was 1.22 (95% CI: 1.15, 1.27) in 2005. The cohort effects decreased monotonically from 1915–1919 to 1990–1994. The change rate fluctuated from 1920–1924 to 1990–1994, including three accelerating and three decelerating decreases. There was a positive spatial autocorrelation in stroke mortality-PM_2.5_ from 1990 to 2015. Hot-spots moved from the northeastern areas to the middle and southwestern areas, whereas cold-spots lay mostly in coastal provinces. Besides the aging process in recent years, stroke mortality-PM_2.5_ had significantly declined from 2005 to 2015 due to socio-economic and healthcare development. Stroke mortality-PM_2.5_ varied substantially among different regions, and cost-effective prevention and control should be implemented more in the middle and southwestern areas of China.

## 1. Introduction

Non-communicable diseases (NCDs), which accounted for approximately 71.3% of global deaths in 2015, are recognized as a major burden on public health [1]. As the second-leading cause of death globally, stroke caused approximately 6.33 million deaths and 118.63 million disability-adjusted life years (DALYs) in 2015, and three-quarters of them occurred in developing countries [2,3]. Stroke ranked the first leading cause of death in China, having caused 1.90 million deaths and 34.81 million DALYs in 2015, and has brought a tremendous health burden to society [4]. It was widely reported that stroke had many risk factors, including high blood pressure, unhealthy diet (high sodium or/and vitamin deficiencies), smoking, lack of exercise, and air pollution [5,6].

Air pollution is a major threat to health worldwide. As an important type of air pollutant, ambient particulate matter pollution (PM_2.5_, particulate matter with an aerodynamic diameter smaller than 2.5 micrometers) was the fourth highest-ranking risk factor for death and caused 80.09 deaths per 100,000 in 2015 in China [7]. Brauer et al., noted that the population-weighted mean concentration of PM_2.5_ in China reached 55 μg/m^3^ in 2013, increasing by 38% from 1990 to 2013. The globally highest value was 194 μg/m^3^, found in Shijiazhuang, the capital of Hebei Province in China [8]. Reducing PM_2.5_ has been listed as one indicator in the sustainable development goals (SDGs). The PM_2.5_ score in the SDGs is the lowest score in China [9]. Therefore, it is urgent to conduct more related research to reveal the burden of disease caused by PM_2.5_, especially in China.

In recent years, an increasing number of studies have focused on the relationship between PM_2.5_ and stroke in China [10,11,12,13]. Song et al., analyzed the health burden of related diseases caused by PM_2.5_ and concluded that PM_2.5_ contributed as much as 40.3% to stroke deaths in 2015 [10]. Based on time-stratified case-crossover, Huang et al., suggested that the association between PM_2.5_ and stroke morbidity/mortality varied with temperature [11]. Liu et al., accessed the spatial and temporal trends of health impacts of stroke, ischemic heart disease and lung cancer caused by PM_2.5_ from 2004 to 2012 through a simple descriptive method only [12]. Liu et al., estimated adult mortality attributable to PM_2.5_ and concluded that in 2013 the premature mortality of stroke-PM_2.5_ reached 1.37 million [13]. However, none of these studies was age-standardized, so the influences of changing demographics were unclear. Furthermore, no study took the uncontrolled cohort and period effects into account. To address such limitations, our study used the data from the Global Burden of Disease (GBD) 2015 to investigate the temporal trends of stroke mortality-PM_2.5_ with the intrinsic estimator (IE) method of age-period-cohort model and analyze the spatial trends and distributions of stroke mortality-PM_2.5_ through global and local spatial autocorrelation.

## 2. Materials and Methods

### 2.1. Data Sources

This study used the latest data from GBD 2015, which provided a comprehensive estimation of 315 causes of death and 79 risk factors or clusters for 195 countries from 1990 to 2015 [7]. Original data, which GBD adapted to estimate mortality and DALY of stroke in China, was mainly from the Cause of Death Reporting System of the Chinese Center for Disease Control and Prevention (CDC), Disease Surveillance Points (DSPs) and the Maternal and Child Surveillance System [6]. Stroke was diagnosed and identified on the basis of the World Health Organization clinical criteria and the 10th version of the International Classification of Diseases (ICD-10) [14]. Stroke mortality-PM_2.5_ in different provinces was age-standardized by the GBD 2015 global age-standard population [14]. In GBD 2015, a Bayesian Hierarchical modeling approach was implemented to estimate the exposure to PM_2.5_, where original data were obtained from the satellite-based estimates, ground measurements and land-use information. To correct the bias problem, a spatially varying flexible framework was used to organize these data. After evaluating the exposure to PM_2.5_, population attributable fractions (PAF) were estimated, which represented how much disease burden was caused by PM_2.5_. Counterfactual analysis was used to calculate the PAF, which assumed that the exposure to other independent risk factors remained unchanged, and then compared the exposure to PM_2.5_ with the theoretical minimum risk exposure level (TMREL) or counterfactual exposure distribution (CED). The methods were described in detail elsewhere [7,15]. When estimating the PAF of PM_2.5_, the relative risks were evaluated by mapping a curve based on the entire range of exposure from published studies and the TMREL, ranging from 2.4 μg/m^3^ to 5.9 μg/m^3^, that was estimated by uniform distribution [7]. Then, the burden of disease attributable to PM_2.5_ was quantitatively evaluated according to the product of PAF and baseline mortality or DALY of disease [5,16].

### 2.2. Methods

Mortality reflects not only death risks experienced by the population in a current year, but also the accumulation of health risks since birth. Common statistical analysis could not decompose these death risks and health risks when estimating mortality [17,18]. The age-period-cohort model (APC) was a prevalently statistical tool to extract information hidden in age-adjusted mortality, which could access age, period, and cohort effects simultaneously [19]. As a classical epidemiological method, however, the practical application of the APC model has been plagued by collinearity problems. Because of the linear relationship among the age, period and cohort, for age = period − cohort, it was hard for the traditional APC model to estimate the net effect for each age, period and cohort effects [19], which could be exhibited by linear Equation (1):

(1) $$Y_{j}=\mu +\alpha \times age_{j}+\beta \times period_{j}+\gamma \times cohort_{j}+\varepsilon_{i}$$

where $Y_{J}$ denoted the response variable—the net effect on stroke mortality-PM_2.5_ for group j, α, β and γ denoted the coefficient of age, period and cohort of APC model, respectively, and μ denoted the intercept of the model. $\varepsilon_{i}$ denoted the residual of the APC model.

Although many different statistical methods have been used to address this issue, there is still no consensus. Based on Kupper and Fu, Yang et al. proposed the intrinsic estimator (IE) algorithm as a solution to un-identification problems associated with the APC model, which could separate age, period and cohort effects from age-specific outcome variables thoroughly and proved to be prior to other algorithms. In the APC model, the design matrix X was an unfilled rank, which must have an eigenvalue that equaled zero. Since $XB_{0}=0$, where $B_{0}$ was a nonzero vector, $B_{0}$ could be treated as a special vector in the solution set of APC model, which was determined by the difference of $b_{1}$ and $b_{2}$, two arbitrary sets of solutions. Thus, we had the formula: $X\left(\hat{b_{1}}-\hat{b_{2}}\right)=X\left(tB_{0}\right)$, where t was an arbitrary real number. In this situation, an arbitrary solution of the APC model could be seen as two parts: $\hat{b}=B+tB_{0}$, where B was the real solution of the APC model, which was the so-called intrinsic estimator (IE). Thus, the IE algorithm had various superiorities, such as estimability, unbiasedness, validity, etc. [20,21,22].

To conduct the IE method of the APC model here, stroke age-specific mortality attributable to PM_2.5_ was recoded into consecutive 5-year periods from 1990 to 2015 and successive 5-year age groups from 25–29 years to 75–79 years in order to estimate age, period, and cohort effects of stroke mortality-PM_2.5_, respectively and separately, which was the fixed form of the IE solution. In this study, we excluded the age groups under 25 years and over 80 years because stroke morbidity and mortality in the group under 25 years was very rare, and individuals over 80 were only recorded as one group in the GBD database, which did not conform to the fixed form of the IE algorithm.

Geographic Information System (GIS) was applied to the spatial analysis of stroke mortality-PM_2.5_ in China. We used global and local spatial autocorrelation analysis to evaluate the degree of clustering from 1990 to 2015. *Z* scores were used to distinguish the spatial clustering, and when the value was larger than 1.96 or smaller than −1.96, the spatial autocorrelation was significant [23]. Global spatial autocorrelation was assessed by the overall spatial distribution at the country level with global Moran’s I statistic, which ranged from −1 to 1, and was used to assess the overall pattern and whether stroke mortality-PM_2.5_ had a spatial cluster trend. As long as there is an area whose neighboring areas tend to be significantly clustered spatially (the high values are clustered by high values, or the lower values are clustered by low values), the Global Moran’s I index will be positive. The Moran’s I index will be negative if the high values tend to be clustered by low values or low values tend to be clustered by high values. The Moran’s I index will be zero if the positive cross-product is offset by the negative cross-product. However, Global spatial autocorrelation could not identify the specific location of clusters significantly at the province level [24]. The Getis-Ord Gi* local statistic, therefore, was implied to supplement this issue. The Getis-Ord Gi* local statistic, also known as Hot Spot Analysis, was further adopted to recognize the location of the clusters with values significantly higher or lower in magnitude at the province level. Based on a null hypothesis of spatial randomness [25], the *Z*-score, the output of the Gi* function, was assigned to each province to recognize the statistical significance of clustering of hot-spots and cold-spots of stroke mortality-PM_2.5_.

In this paper, APC analyses were implemented using the Stata 12.0 software (StataCorp, College Station, TX, USA). Deviance, Akaike Information Criterion (AIC) and Bayesian Information Criterion (BIC) were used to check the degree of model fitting. The spatial autocorrelation was analyzed by ArcGIS 10.2.2 (ESRI Inc., Redlands, CA, USA), 999 permutations were set for randomization, and *p* < 0.05 was considered statistically significant.

## 3. Results

**Figure 1:**
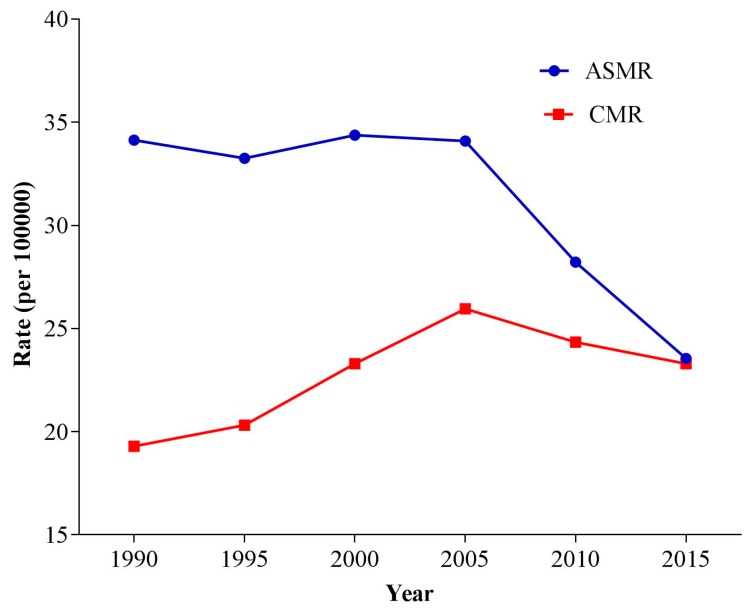
Trends of the crude mortality rates (CMRs) and age-standardized mortality rates (ASMRs) for stroke attributable to PM_2.5_ from 1990–2015.

Trends of the crude mortality rates (CMRs) and age-standardized mortality rates (ASMRs) for stroke attributable to PM_2.5_ from 1990 to 2015 are shown in Figure 1. In general, ASMRs were higher than CMRs for the period from 1990 to 2015 and tended to be equal in 2015. The CMRs experienced a remarkable increase from 1990 to 2005, then slightly decreased from 2005 to 2015. The ASMRs for stroke attributable to PM_2.5_ showed a general downward trend since 1990 that decreased slightly from 1990 to 2005 and decreased more profoundly after 2005. 

### 3.1. The Variation of Age, Period, and Cohort on Stroke Mortality-PM_2.5_

**Figure 2:**
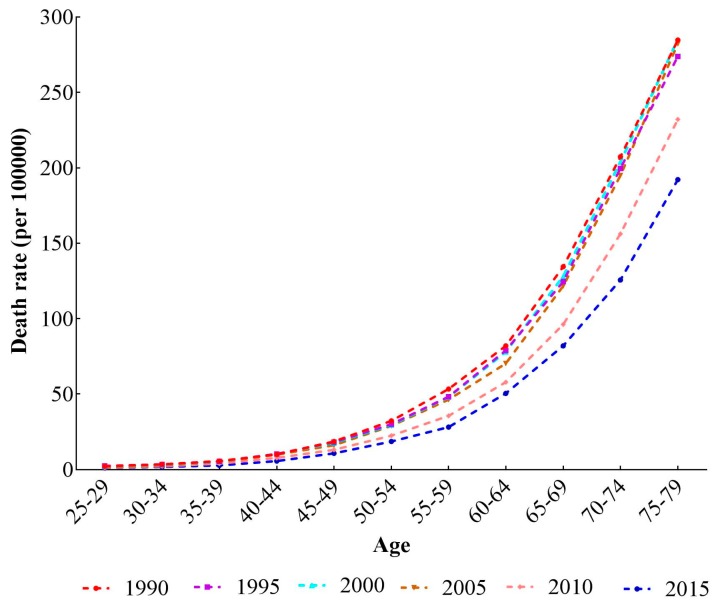
Age-specific stroke mortality-PM_2.5_ in China from 1990–2015.

Trends of the age-specific stroke mortality-PM_2.5_ in 1990, 1995, 2000, 2005, 2010 and 2015 are shown in Figure 2. According to Figure 2, stroke mortality-PM_2.5_ approximately followed an exponential distribution with age. Compared with the younger population, elderly people suffered a higher mortality rate. In addition, we found a declining trend among stroke mortality-PM_2.5_ in six periods, from 1990 to 2015, and the elder population decreased more than young people in absolute mortality values.

**Figure 3:**
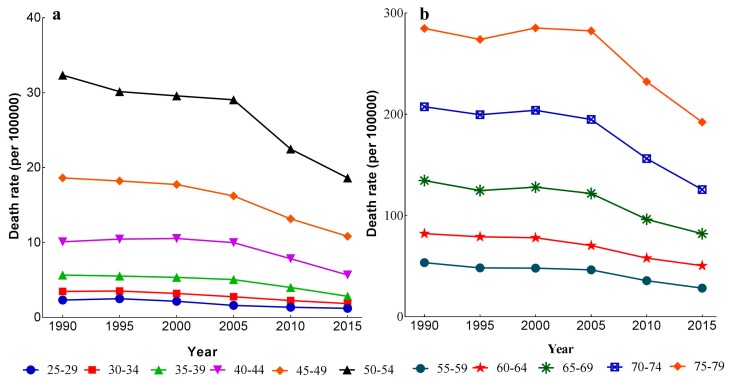
Stroke mortality-PM_2.5_ among different age groups from 1990 to 2015. (**a**) 25–54 years old; (**b**) 55–79 years old.

The variation trends of stroke mortality-PM_2.5_ of different age groups from 1990 to 2015 are shown in Figure 3. Figure 3a shows the variation of mortality rates in the group aged 25–54 years old, and Figure 3b shows the mortality variation in the group aged 55–79 years old. From these two figures, we can see that the mortality rates of all age groups decreased with the advance of time periods, except for the 25–29, 30–34, and 40–44 age groups, which showed an upward trend from 1990 to 1995. Stroke mortality-PM_2.5_ increased significantly from 1990 to 2015 with age. The mortality rates of older age groups in Figure 3b were much higher than those of younger age groups in Figure 3a.

**Figure 4:**
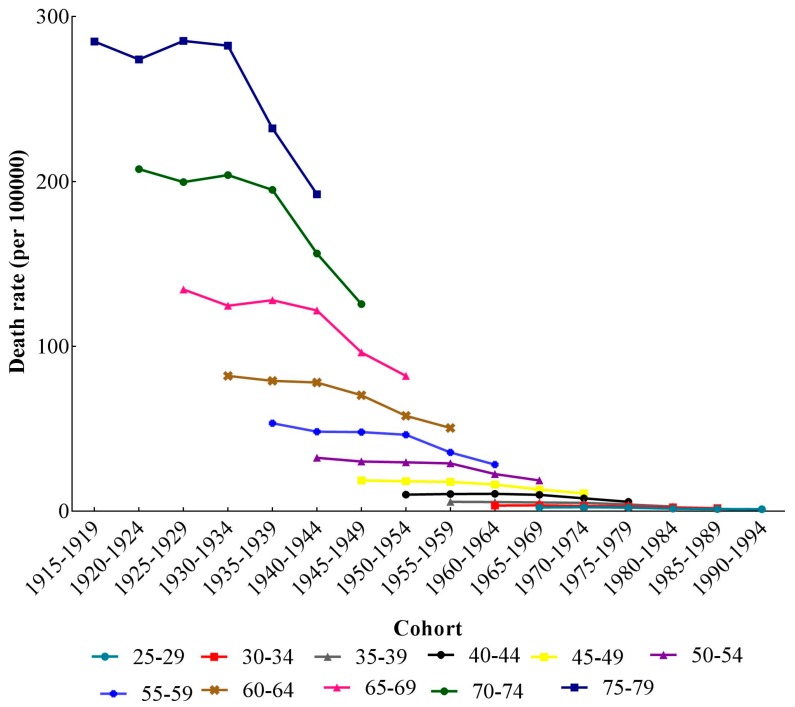
Cohort-based variation of age-specific stroke mortality-PM_2.5_.

The cohort-based variation of age-specific stroke mortality-PM_2.5_ is shown in Figure 3. For all age groups, the mortality rates decreased continuously with the year of birth. Moreover, the stroke mortality-PM_2.5_ of elder groups had larger declines with time period. In the same age group, there was a big difference in the mortality rates of people born in different cohorts. In general, stroke mortality-PM_2.5_ for younger groups was lower than that in the older groups. The cohort effects span almost 100 years in Figure 4. Since age and period effects on the mortality were confounded, we failed to find independent cohort effects.

### 3.2. The Results of Stroke Mortality-PM_2.5_ from the IE Method of APC Model Analysis

**Figure 5:**
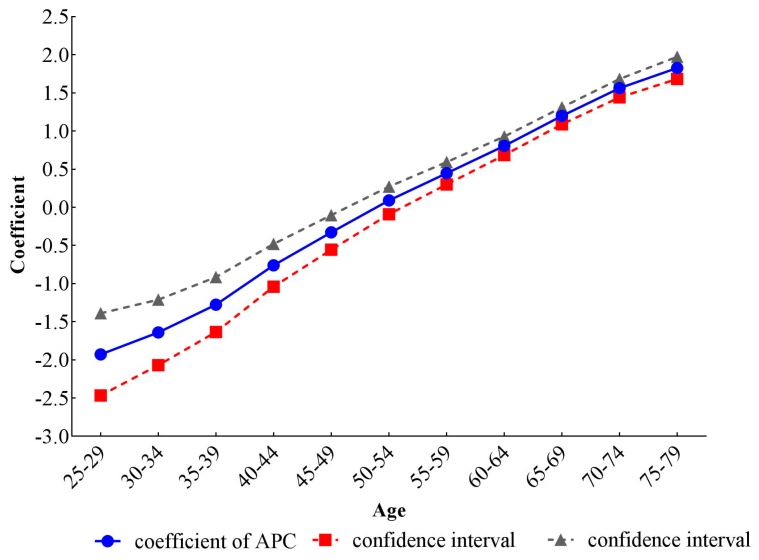
Age effects on stroke mortality PM_2.5_ in China.

**Figure 6:**
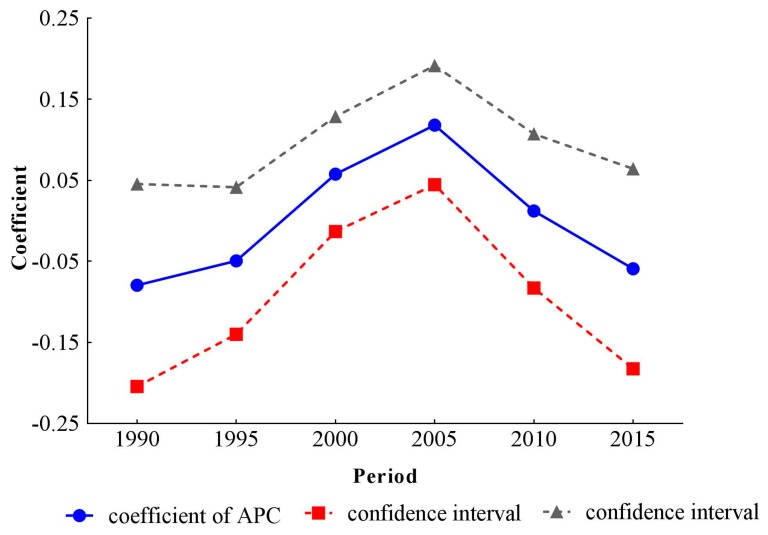
Period effects on stroke mortality PM_2.5_ in China.

**Figure 7:**
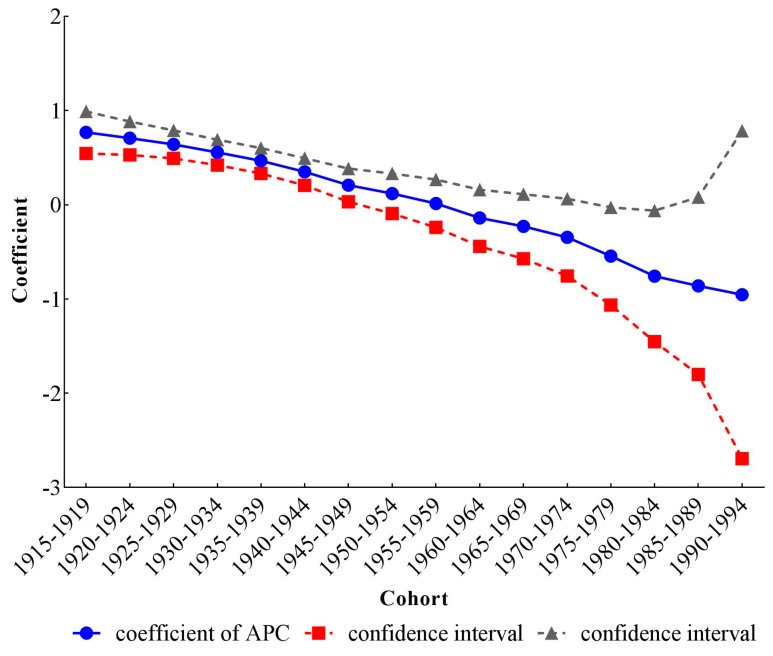
Cohort effects on stroke mortality PM_2.5_ in China.

The estimated coefficients, significance levels, standard errors and relative risk (RR) of age, period and cohort effects are shown in Table 1. For the calculation of RR, we chose the first group of 25–29 years old in age, the 1990 time period and the 1915–1919 cohort as reference groups, and we then calculated the difference (d) between other groups and reference groups, respectively. The exponential value of d denoted the mortality RR of a particular age, period and birth cohort relative to reference groups. To intuitively describe the trends of three different temporal effects on stroke mortality-PM_2.5_, line graphs of age, period and cohort coefficients and their 95% confidence intervals were drawn in Figure 5, Figure 6 and Figure 7.

After controlling the period and cohort effects, the age coefficients on stroke mortality-PM_2.5_ continuously increased from −1.93 (−2.47, −1.39) in the group aged 25–29 to 1.83 (1.68, 1.97) in the group aged 75–79. The mortality risk in the group aged 75–79 was 42.85 (28.79, 63.43) times greater than that in the group aged 25–29. The coefficients for period effects showed a reversed V shape, which increased from −0.08 (−0.20, 0.05) in 1990 to 0.12 (0.04, 0.19) in 2005, then decreased to −0.06 (−0.18, 0.06) in 2015. Compared with RR in 1990, the RR in 2005 and 2015 was 1.22 (1.15, 1.27) and 1.02 (1.01, 1.02), respectively, indicating that the RR of stroke mortality-PM_2.5_ was the largest in 2005. From the cohort effects, we could see that the earlier birth cohorts would experience a higher mortality, while the later cohorts experienced a lower mortality. The coefficient decreased by 1.72 (0.20, 3.25) from the cohort 1915–1919 to 1990–1994. The RR of stroke mortality-PM_2.5_ also showed a monotonically decreasing trend, dropping to 0.18 (0.04, 0.82) for the cohort 1990–1994.

**Figure 8:**
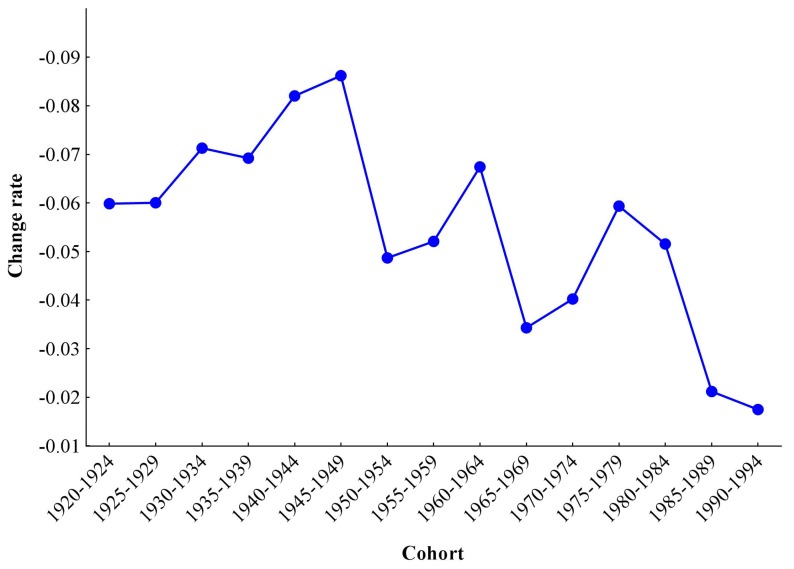
Change rate of cohort effects on stroke mortality PM-2.5 in China.

To interpret the variation of cohort effects, we used the RR of the 1915–1919 cohort as a reference, and then in turn we made the RRs of other cohorts subtract this reference RR to get the change rate according to the principle of numerical differentiation. The value of the change rate was positive when mortality risk increased, while it was negative when RR decreased and 0 when RR remained unchanged. The Y-axis was set to reverse, and since the values were all negative, the line rising meant the accelerated decline of RR of stroke mortality-PM_2.5_; the line declined when the change rate decelerated and was paralleled when the change rate declined uniformly. As shown in Figure 8, the change rate of cohort effects fluctuated from 1920–1924 to 1990–1994. 

The change rate could be divided into six stages, including three accelerating decreases and three decelerating decreases. The first accelerating decrease was from 1920–1924 to 1945–1949, which accelerated by 0.03 (except for 1935–1939 cohort when it decelerated compared with the previous cohort); the second accelerating decrease ran from 1950–1954 to 1960–1964 and accelerated by 0.02; and the third accelerating decrease was from 1965–1969 to 1975–1979, and then it accelerated by 0.03. The three decelerating decreases were from 1945–1949 to 1950–1954, 1960–1964 to 1965–1969, and 1975–1979 to 1990–1994, which decelerated by 0.04, 0.03, 0.04, respectively.

### 3.3. The Results of Spatial Autocorrelation

**Figure 9:**
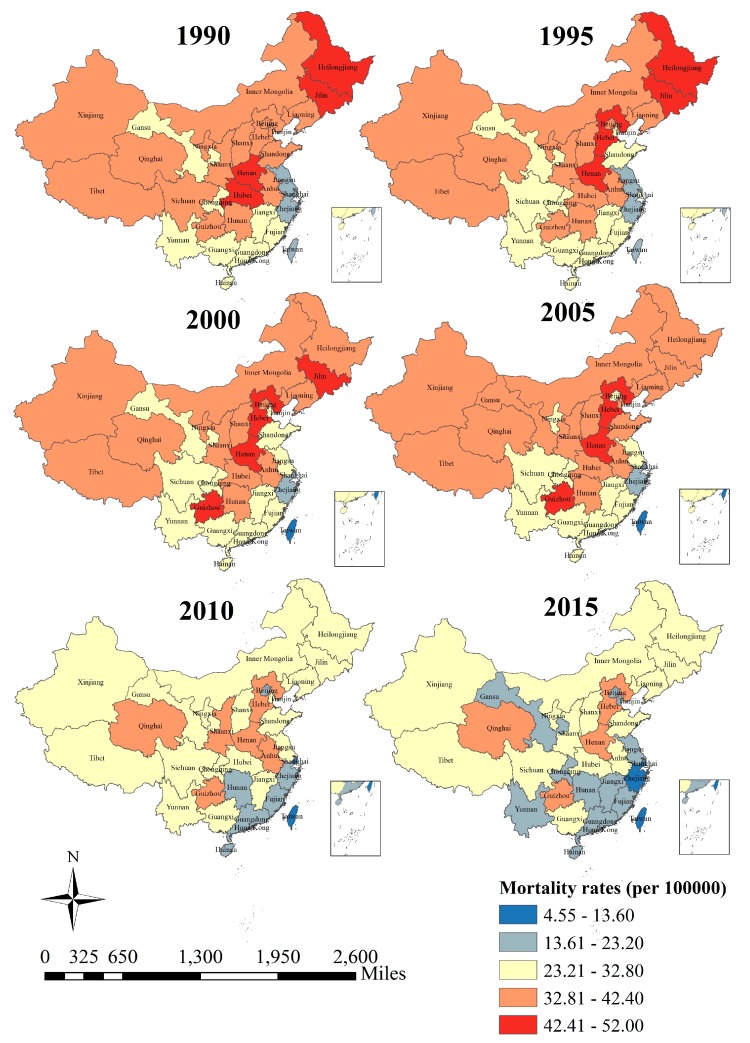
The description of stroke mortality-PM_2.5_ by province in 1990–2015, China.

To reveal the spatial distribution models of stroke mortality attributable to PM_2.5_, we first described its spatial distribution simply through the real value. The description of stroke mortality-PM_2.5_ by province is shown in Figure 9.

Three main rules could be found from Figure 9. First, an outstanding inter-province difference could be found from a single period. In all six periods from 1990 to 2015, the stroke mortality-PM_2.5_ was lower in the southeast and higher in northern and western China. Second, an obvious decrease in stroke mortality-PM_2.5_ could be found from the perspective of social change from 1990 to 2015, such that a later period would exhibit a relatively lower stroke mortality-PM_2.5_ and an earlier period would display a relatively higher stroke mortality-PM_2.5_, such as the comparison of stroke mortality-PM_2.5_ between 1990 and 2015. Third and last, although almost all of these provinces have achieved great progress during these 25 years, the declining speed of stroke mortality-PM_2.5_ was not the same across different provinces. 

For instance, for the period from 1990–2015, the stroke mortality-PM_2.5_ has decreased rapidly in Heilongjiang and Jilin provinces, while Qinghai Province has achieved little progress in the prevention of stroke mortality-PM_2.5_. Since simple description could not make clear the associations among provinces, especially those between a specific province and some other adjacent provinces, we then conducted a spatial autocorrelation analysis to uncover it.

**Figure 10:**
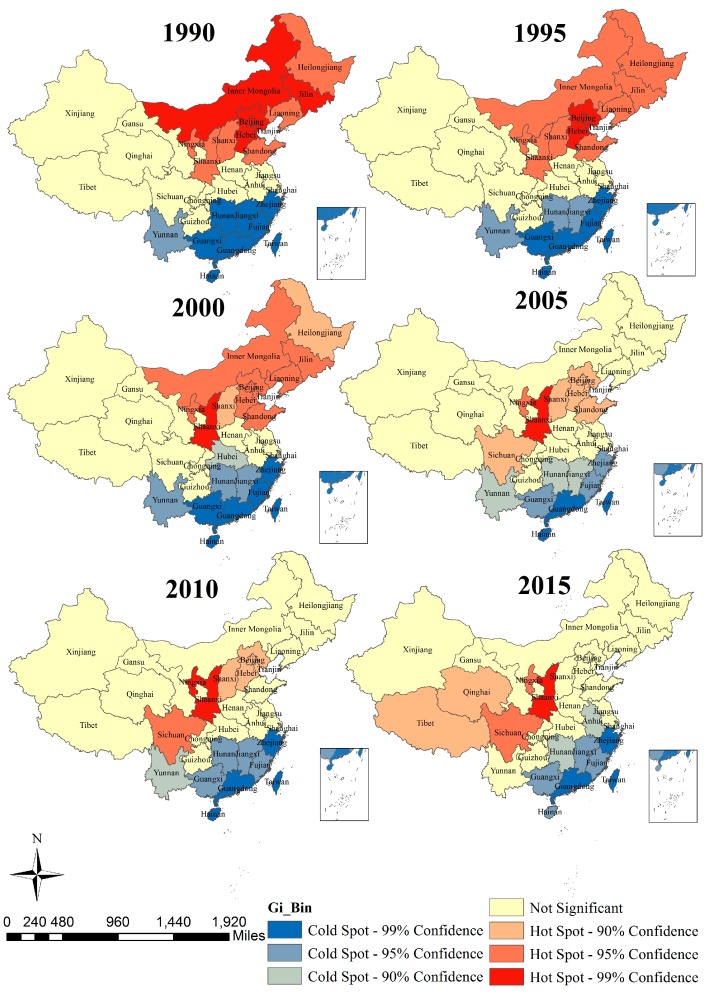
Local spatial autocorrelation of stroke mortality PM-2.5 in 1990–2015, China.

The global Moran’s I index and its *Z* scores and *p* values in stroke mortality-PM_2.5_ from 1990 to 2015 at the country level are displayed in Table 2. We can see that all of the *Z* scores in this study were greater than 1.96, and *p* values were less than 0.05, which indicated that the spatial correlation on stroke mortality-PM_2.5_ was statistically significant. All values of Moran’s I were greater than 0, which suggested that there were positive autocorrelations on the mortality rates. In all six periods, the slight decline of Moran’s I from 0.55 in 1990 to 0.39 in 2015 indicated a decline clustering tendency of stroke mortality-PM_2.5_. Our investigation on the Getis-Ord Gi* statistic gave more clues on core hot-spot/cold-spot clusters. Primary (Gi* *Z*-score > 2.58 SD), secondary (1.96 SD < Gi* *Z*-score ≤ 2.58 SD), and tertiary (1.64 SD < Gi* *Z*-score ≤ 1.96 SD) intensity clusters from 1990 to 2015 at the province level are displayed in Figure 10. 

Hot-spots are mainly located in China’s northeastern regions from 1990 to 2000, such as Inner Mongolia, Jilin, Heilongjiang, Hebei, Beijing, Shanxi, and Shaanxi, but these hotspots gradually moved to central and western provinces after 2000 (Shaanxi, Sichuan, Qinghai and Tibet). Cold-spots were located in the southern littoral regions of China, such as Zhejiang, Fujian, Guangdong, Hong Kong and Taiwan.

## 4. Discussion

This is the first study to examine net age-, period-, and cohort-specific effects separately on stroke mortality-PM_2.5_ through the IE method of the APC model and the first to examine the presence of spatial clusters of stroke mortality-PM_2.5_ in China at both the national and provincial levels using age-standardized data. 

Age was reported as the most important risk factor for stroke [26]. In this study, after controlling period and cohort effects, stroke mortality risk attributable to PM_2.5_ showed an exponential growth trend with age, and the group aged 75–79 years suffered the largest mortality risk, which was consistent with the finding of another study conducted by Wang [26]. The increasing trend observed in age effects was mainly influenced by biological factors. Elderly people had poor immune function and were vulnerable to the impact of PM_2.5_, which would cause lung infection and then lead to the occurrence of stroke [27]. In addition, elder groups had fewer opportunities to take preventive measures because of weak health awareness, so they might experience more exposure to low-hygiene environments [28,29]. All these factors would lead to higher mortality risk among older groups. It should be noted that the trends between the CMRs and ASMRs during 1990–2015 were different. In our study, the CMRs attributable to PM_2.5_ increased significantly during the period 1990–2015, while the ASMRs showed a downward trend, which was similar to the results of the stroke mortality study [26]. This difference was mainly related to China’s aging progress [30]. China has the largest population in the world, and the number and the proportion of the population aged 60 and over rose rapidly from 1980 to 2010 because of the decreasing fertility rate and increasing life expectancy [31]. The “Healthy China 2030” plan noted that the life expectancy in China was 76.34 in 2015 and was expected to be 79 in 2030 [32]. In Table 1, we can see that the greatest mortality risk occurred in people aged 60 and over. Thus, the increasing proportion of the elderly would result in the growth of the crude mortality. The ASMRs were age-standardized by GBD 2015 global age-standard population, so they were equal to the CMRs in 2015 [14].

Period effects suggested the immediate effects of factors on disease morbidity and mortality [33]. In our study, period effects on stroke mortality-PM_2.5_ increased by 22% from 1990 to 2005, then decreased from 2005 to 2015. This result was not exactly consistent with the study conducted by Liu, which indicated that the stroke deaths increased from 2005 to 2009 and then decreased from 2009 to 2012 [12]. This could be explained by the fact that they only considered excess deaths, while we considered the impacts of population growth, aging and cohort effects. Economy, environment and policy factors could be used to explain the increasing trend from 1990 to 2005. During these periods, pollution had gradually deteriorated environmental quality due to the rapid growth of the economy and urbanization, and the healthcare system was relatively underdeveloped, which increased the burden of stroke attributable to PM_2.5_ [34,35]. In contrast, the mortality risk from 2005 to 2015 decreased, which was caused by the combined effects of improvements in environmental quality and healthcare conditions [12]. The strengthened particulate matter emission standard for power plants in 2003 and the 11th Five-Year Plan in 2006 proposed stricter energy conservation and emission reduction policies, which led to a huge improvement of air pollution [36]. The improvements in the air conditions reduced the health damage caused by PM_2.5_ [10,36]. In addition, a significant improvement of medical technology also played an important role in the declining mortality risk from 2005 to 2015. After the new medical reform in 2009, the healthcare system in China was reformed, which helped most stroke patients receive early diagnosis and effective treatment [37]. Implementation of the new rural cooperative medical care also made medical services more available for people living in rural areas, which greatly reduced case fatality [38]. 

The cohort effects on stroke mortality risk-PM_2.5_ showed a significant decreasing trend. Compared with later cohorts, earlier cohorts experienced a higher mortality. One potential reason was that earlier cohorts could not get adequate nutrition in their childhood, and therefore, they were not protected by a strong immune system like the later cohorts. [39]. In addition, education and health awareness also played a critical role. Because the level of education was low in early stages, earlier cohorts had weak health awareness, so they realized neither the occurrence of stroke nor the damage of PM_2.5_ to human health [40]. As a result, stroke could not be prevented effectively or treated in a timely fashion among earlier cohorts. The change rate of cohort effects on stroke mortality-PM_2.5_ fluctuated from 1920–1924 to 1990–1994. Three accelerating and three decelerating decreases were noted when interpreting the variation of the change rate. The accelerating decreases indicated improvements in the situation of stroke mortality risk attributable to PM_2.5_, and the decelerating decreases represented deterioration. The environmental change and childhood experience had a lasting impact on the stroke mortality risk attributable to PM_2.5_ for the rest of their life [41]. From 1920 to 1949, medical and public health services began to form, and air pollution was not serious due to the low level of industrial development, which greatly promoted the prevention and treatment of stroke in their childhood [42]. The fluctuation from 1950 to 1980 mainly involved the air pollution caused by industrial development and the status of healthcare services. From 1950 to 1959, since the 1st Five-Year Plan was carried out, the heavy industry rate increased from 26.4 to 48.4%, which caused a huge burden on the environment [43], and this cohort also experienced the latter natural disaster in 1959, which led to maldevelopment or other health risks, and was easier to be invaded by stroke. For 1960–1964 cohort, they were born in the era of the economy adjustment after the Great Famine, when they could get more nutrition and have strong immune to resist disease. Another reason might be that the industrial development in this period was stagnant [44]. During the Great Proletarian Cultural Revolution from 1966 to 1976, the development of medical and healthcare services was severely hampered [19]. After the policy of reform and opening in 1978, China’s economy experienced a period of rapid development. During this time, industrialization, urbanization and population growth made air pollution worse [36,45]. All in all, however, social development, especially the development of healthcare during the entirety of the 20th century, made the stroke mortality-PM_2.5_ constantly decline.

Stroke mortality-PM_2.5_ in northern China was higher than that in the south. The global spatial autocorrelation analysis showed a positive autocorrelation of stroke mortality-PM_2.5_ in China. Moreover, the comparison of the six periods also suggested that the distribution scope of hot-spot clusters had been gradually shrinking from 1990 to 2015, which implied that the status of stroke mortality-PM_2.5_ has gradually improved. According to Figure 9 and Figure 10, we can see that the hot-spots move from the western and northeastern areas to the middle and southwestern areas, which can be deciphered by the distinct improvement speed of different areas. In 2015, high mortality rates were mainly concentrated in Shaanxi, Ningxia, Qinghai, Tibet and Sichuan, and low mortality rates were located in southeast coastal areas (such as Zhejiang, Fujian, Guangdong, Hainan, etc.), which were consistent with the spatial distributions of stroke mortality and PM_2.5_ in China [12]. Stroke mortality-PM_2.5_ was determined by baseline mortality (influenced by living condition and healthcare services), population, and PAF (which depends on PM_2.5_ concentrations) [10]. According to previous studies, the spatial pattern of PM_2.5_ concentrations in China has remained stable over time [46]. As a result, the reduction of hot-spots from 1990 to 2015 mainly depended on stroke mortality rates of different regions. The techniques for diagnosis and treatment of stroke have improved profoundly, and stroke mortality was found to be declining throughout these 25 years [26]. For provinces with low PM_2.5_ concentrations, like Inner Mongolia, Heilongjiang and Jilin, a decrease of stroke mortality could significantly improve the status quo of stroke mortality-PM_2.5_. Provinces like Zhejiang, Fujian, and Guangdong had lower PM_2.5_ concentrations and lower stroke mortality, as well as a decline in PAF, so there existed cold-spots of stroke mortality-PM_2.5_ among these areas [10,12]. Our finding was not exactly consistent with previous studies conducted by Liu and Liu, mainly because they used the number of deaths [12,13]. Since the population structures in different provinces were not the same, the deaths could not be compared directly. Consequently, our study adopted an age-standardized rate to analyze the spatial pattern of stroke mortality-PM_2.5_. The spatial variation of stroke mortality-risk should be considered in attempts to set emission reduction targets and solutions to the problem of inequality in medical service. Hot-spot areas should be emphatically considered to be the focus for stroke prevention.

Nevertheless, our study had some limitations. First, all data in the present study were extracted from the GBD 2015, which estimated the burden of diseases systematically in different countries and regions from a global perspective. Although many methods were used to reduce bias, including misclassification corrections, incompleteness and redistribution of garbage codes, it was difficult to thoroughly avoid inaccuracy [47]. Therefore, considering the data we used were not observed but estimated, our results from modeling estimates of stroke mortality-PM_2.5_ should be treated carefully. Second, due to the fixed pattern of the IE method, the age groups over 80 were excluded in this study. However, in our study, stroke mortality-PM_2.5_ increases exponentially with age, so the groups aged 80 and older were supposed to have even higher mortality risk compared with the younger age groups. Third, because the mortality data we used were insufficient, our study lacked analysis of the comparison of stroke-mortality-PM_2.5_ between urban and rural areas. Therefore, more analyses of stroke mortality-PM_2.5_ in urban and rural areas need to be conducted in the future.

## 5. Conclusions

In summary, we evaluated the temporal and spatial tendencies in stroke mortality rates attributable to PM_2.5_ in China during 1990–2015. Through the IE method of APC analysis, stroke mortality-PM_2.5_ increased exponentially with age, first increased and then decreased with time period, and decreased continuously with cohort. There was a positive spatial autocorrelation in stroke mortality-PM_2.5_ at the country level in China from 1990 to 2015. Hot-spots moved from northeastern areas to the middle and southwestern areas, whereas cold-spots were mainly located in provinces along the southern coastal regions of China. Besides the aging process in recent years, stroke mortality-PM_2.5_ significantly declined from 2005 to 2015 due to socio-economic and healthcare development. Stroke mortality-PM_2.5_ varied substantially among different regions. Therefore, some effective measures should be taken to enhance the protection of the high-risk population from PM_2.5_ and to strengthen the governance of the middle and southwestern areas of China to reduce the burden of disease caused by PM_2.5_.

## Figures and Tables

**Table 1:** Age-period-cohort (APC) model analysis results of stroke mortality-PM_2.5_ in China.

Variables	Coef	95% CI	S.E.	RR	95% CI
Intercept	3.13	(2.98, 3.28)	0.08		
Age					
25–29	−1.93 ***	(−2.47, −1.39)	0.27	1.00	
30–34	−1.64 ***	(−2.07, −1.21)	0.22	1.33	(1.20, 1.49)
35–39	−1.28 ***	(−1.64, −0.92)	0.18	1.92	(1.60, 2.29)
40–44	−0.76 ***	(−1.04, −0.48)	0.14	3.22	(2.48, 4.18)
45–49	−0.33 ***	(−0.56, −0.10)	0.12	4.96	(3.63, 6.75)
50–54	0.09	(−0.09, 0.27)	0.09	7.54	(5.26, 10.80)
55–59	0.45 ***	(0.30, 0.59)	0.07	10.75	(7.24, 15.96)
60–64	0.81 ***	(0.69, 0.93)	0.06	15.43	(10.18, 23.57)
65–69	1.20 ***	(1.09, 1.31)	0.06	22.87	(14.88, 35.16)
70–74	1.56 ***	(1.44, 1.68)	0.06	32.87	(21.54, 49.90)
75–79	1.83 ***	(1.68, 1.97)	0.07	42.85	(28.79, 63.43)
Period					
1990	−0.08	(−0.20, 0.05)	0.06	1.00	
1995	−0.05	(−0.14, 0.04)	0.05	1.03	(0.99, 1.06)
2000	0.06	(−0.01, 0.13)	0.04	1.15	(1.08, 1.21)
2005	0.12 **	(0.04, 0.19)	0.04	1.22	(1.15, 1.27)
2010	0.01	(−0.08, 0.11)	0.05	1.10	(1.06, 1.13)
2015	−0.06	(−0.18, 0.06)	0.06	1.02	(1.01, 1.02)
Cohort					
1915–1919	0.77 ***	(0.55, 0.99)	0.11	1.00	
1920–1924	0.71 ***	(0.53, 0.88)	0.09	0.94	(0.90, 0.98)
1925–1929	0.64 ***	(0.49, 0.79)	0.08	0.88	(0.82, 0.94)
1930–1934	0.56 ***	(0.42, 0.69)	0.07	0.81	(0.74, 0.88)
1935–1939	0.47 ***	(0.33, 0.6)	0.07	0.74	(0.68, 0.80)
1940–1944	0.35 ***	(0.21, 0.49)	0.07	0.66	(0.61, 0.71)
1945–1949	0.21	(0.03, 0.38)	0.09	0.57	(0.54, 0.59)
1950–1954	0.12	(−0.09, 0.33)	0.11	0.52	(0.52, 0.53)
1955–1959	0.01	(−0.24, 0.27)	0.13	0.47	(0.45, 0.49)
1960–1964	−0.14	(−0.44, 0.16)	0.15	0.40	(0.37, 0.44)
1965–1969	−0.23	(−0.57, 0.11)	0.17	0.37	(0.33, 0.41)
1970–1974	−0.35	(−0.76, 0.07)	0.21	0.33	(0.27, 0.40)
1975–1979	−0.54 *	(−1.06, −0.03)	0.26	0.27	(0.20, 0.36)
1980–1984	−0.76 *	(−1.45, −0.06)	0.36	0.22	(0.14, 0.35)
1985–1989	−0.86	(−1.80, 0.08)	0.48	0.20	(0.10, 0.40)
1990–1994	−0.95	(−2.70, 0.79)	0.89	0.18	(0.04, 0.82)
Deviance	0.81				
AIC	5.94				
BIC	−150.02				

Note: * *p* < 0.05; ** *p* < 0.01; *** *p* < 0.001; Coef.: Coefficient; S.E.: Standard error; RR: Relative risk; CI: Confidence interval; AIC: Akaike Information Criterions; BIC: Bayesian Information Criterions.

**Table 2:** Global spatial autocorrelation of stroke mortality PM_2.5_ in 1990–2015, China.

Year	Moran’s I	*Z* Score	*p*
1990	0.55	7.36	<0.001
1995	0.53	7.15	<0.001
2000	0.54	7.34	<0.001
2005	0.44	6.12	<0.001
2010	0.44	6.09	<0.001
2015	0.39	5.35	<0.001

## References

[1] Mendis S., Davis S., Norrving B. (2015). Organizational update: The World Health Organization Global Status Report on Noncommunicable Diseases 2014; one more landmark step in the combat against stroke and vascular disease. Stroke.

[2] Wang H., Naghavi M., Allen C., Barber R.M., Bhutta Z.A., Carter A., Casey D.C., Charlson F.J., Chen A.Z., Coates M.M. (2016). Global, regional, and national life expectancy, all-cause mortality, and cause-specific mortality for 249 causes of death, 1980–2015: A systematic analysis for the Global Burden of Disease Study 2015. Lancet.

[3] Kassebaum N.J., Arora M., Barber R.M., Bhutta Z.A., Brown J., Carter A., Casey D.C., Charlson F.J., Coates M.M., Coggeshall M. (2016). Global, regional, and national disability-adjusted life-years (DALYs) for 315 diseases and injuries and healthy life expectancy (HALE), 1990–2015: A systematic analysis for the Global Burden of Disease Study 2015. Lancet.

[4] (2017). Global Burden of Diseases Study 2015. Global Burden of Diseases Study 2015 (GBD 2015) by Location, Cause, and Risk Factors.

[5] Feigin V.L., Roth G.A., Naghavi M., Parmar P., Krishnamurthi R., Chugh S., Mensah G.A., Norrving B., Shiue I., Ng M. (2016). Global burden of stroke and risk factors in 188 countries, during 1990–2013: A systematic analysis for the Global Burden of Disease Study 2013. Lancet Neurol..

[6] Zhou M., Wang H., Zhu J., Chen W., Wang L., Liu S., Li Y., Wang L., Liu Y., Yin P. (2016). Cause-specific mortality for 240 causes in China during 1990–2013: A systematic subnational analysis for the Global Burden of Disease Study 2013. Lancet.

[7] Forouzanfar M.H., Afshin A., Alexander L.T., Anderson H.R., Bhutta Z.A., Biryukov S., Brauer M., Burnett R., Cercy K., Charlson F.J. (2016). Global, regional, and national comparative risk assessment of 79 behavioural, environmental and occupational, and metabolic risks or clusters of risks, 1990–2015: A systematic analysis for the Global Burden of Disease Study 2015. Lancet.

[8] Brauer M., Freedman G., Frostad J., van Donkelaar A., Martin R.V., Dentener F., van Dingenen R., Estep K., Amini H., Apte J.S. (2016). Ambient Air Pollution Exposure Estimation for the Global Burden of Disease 2013. Environ. Sci. Technol..

[9] Lim S.S., Allen K., Bhutta Z.A., Dandona L., Forouzanfar M.H., Fullman N., Gething P.W., Goldberg E.M., Hay S.I., Holmberg M. (2016). Measuring the health-related Sustainable Development Goals in 188 countries: A baseline analysis from the Global Burden of Disease Study 2015. Lancet.

[10] Song C., He J., Wu L. (2017). Health burden attributable to ambient PM2.5 in China. Environ. Pollut..

[11] Huang F., Luo Y., Guo Y. (2016). Particulate Matter and Hospital Admissions for Stroke in Beijing, China: Modification Effects by Ambient Temperature. J. Am. Heart Assoc..

[12] Liu M., Huang Y., Ma Z., Jin Z., Liu X., Wang H., Liu Y., Wang J., Jantunen M., Bi J. (2017). Spatial and temporal trends in the mortality burden of air pollution in China: 2004–2012. Environ. Int..

[13] Liu J., Han Y., Tang X., Zhu J., Zhu T. (2016). Estimating adult mortality attributable to PM_2.5_ exposure in China with assimilated PM_2.5_ concentrations based on a ground monitoring network. Sci. Total Environ..

[14] Feigin V.L., Krishnamurthi R.V., Parmar P., Norrving B., Mensah G.A., Bennett D.A., Barker-Collo S., Moran A.E., Sacco R.L., Truelsen T. (2015). Update on the Global Burden of Ischemic and Hemorrhagic Stroke in 1990–2013: The GBD 2013 Study. Neuroepidemiology.

[15] Roth G.A., Johnson C.O., Nguyen G., Naghavi M., Feigin V.L., Murray C.J., Forouzanfar M.H., Vos T. (2015). Methods for Estimating the Global Burden of Cerebrovascular Diseases. Neuroepidemiology.

[16] Lim S.S., Vos T., Flaxman A.D., Danaei G., Shibuya K., Adair-Rohani H., Amann M., Anderson H.R., Andrews K.G., Aryee M. (2012). A comparative risk assessment of burden of disease and injury attributable to 67 risk factors and risk factor clusters in 21 regions, 1990–2010: A systematic analysis for the Global Burden of Disease Study 2010. Lancet.

[17] Wang P., Xu C., Yu C. (2014). Age-period-cohort analysis on the cancer mortality in rural China: 1990–2010. Int. J. Equity Health.

[18] Wang J., Bai Z., Wang Z., Yu C. (2016). Comparison of Secular Trends in Cervical Cancer Mortality in China and the United States: An Age-Period-Cohort Analysis. Int. J. Environ. Res. Public Health.

[19] Robertson C., Gandini S., Boyle P. (1999). Age-period-cohort models: A comparative study of available methodologies. J. Clin. Epidemiol..

[20] Chen X., Wang P. (2014). Dynamic changes of social transformation and national health in China. Chin. J. Popul. Sci..

[21] Yang Y., Fu W., Land K.C. (2004). A methodological comparison of age-period-cohort models: The intrinsic estimator and conventional generalized linear models. Sociol. Methodol..

[22] Li C., Yu C., Wang P. (2015). An age-period-cohort analysis of female breast cancer mortality from 1990–2009 in China. Int. J. Equity Health.

[23] Naves L.A., Porto L.B., Rosa J.W., Casulari L.A., Rosa J.W.C. (2015). Geographical information system (GIS) as a new tool to evaluate epidemiology based on spatial analysis and clinical outcomes in acromegaly. Pituitary.

[24] Wang W., Ying Y., Wu Q., Zhang H., Ma D., Xiao W. (2015). A GIS-based spatial correlation analysis for ambient air pollution and AECOPD hospitalizations in Jinan, China. Respir. Med..

[25] Bao J., Yang X., Zhao Z., Bao J., Yang X., Zhao Z., Wang Z., Yu C., Li X. (2015). The Spatial-Temporal Characteristics of Air Pollution in China from 2001–2014. Int. J. Environ. Res. Public Health.

[26] Wang Z., Hu S., Sang S., Luo L., Yu C. (2017). Age-Period-Cohort Analysis of Stroke Mortality in China: Data From the Global Burden of Disease Study 2013. Stroke.

[27] Qian Y., Zhu M., Cai B. (2013). Epidemiological evidence on association between ambient air pollution and stroke mortality. J. Epidemiol. Community Health.

[28] Hansen S., Baptiste K.E., Fjeldborg J., Horohov D.W. (2015). A review of the equine age-related changes in the immune system: Comparisons between human and equine aging, with focus on lung-specific immune-aging. Ageing Res. Rev..

[29] Smith C.J., Lawrence C.B., Rodriguez-Grande B., Kovacs K.J., Pradillo J.M., Denes A. (2013). The immune system in stroke: Clinical challenges and their translation to experimental research. J. Neuroimmune Pharmacol..

[30] Kanasi E., Ayilavarapu S., Jones J. (2016). The aging population: Demographics and the biology of aging. Periodontoloy.

[31] Lutz W., Sanderson W., Scherbov S. (2008). The coming acceleration of global population ageing. Nature.

[32] The Lancet (2016). The best science for achieving Healthy China 2030. Lancet.

[33] Wang Z., Wang J., Bao J., Gao X., Yu C., Xiang H. (2016). Temporal Trends of Suicide Mortality in Mainland China: Results from the Age-Period-Cohort Framework. Int. J. Environ. Res. Public Health.

[34] Li X., Song J., Lin T., Dixon J., Zhang G., Ye H. (2016). Urbanization and health in China, thinking at the national, local and individual levels. Environ. Health.

[35] Zeng W., Zhen J., Feng M., Campbell S.M., Finlayson A.E., Godman B. (2014). Analysis of the influence of recent reforms in China: Cardiovascular and cerebrovascular medicines as a case history to provide future direction. J. Comp. Eff. Res..

[36] Jin Y., Andersson H., Zhang S. (2016). Air Pollution Control Policies in China: A Retrospective and Prospects. Int. J. Environ. Res. Public Health.

[37] Wang C., Rao K., Wu S., Liu Q. (2013). Health care in China: Improvement, challenges, and reform. Chest J..

[38] Li C., Hou Y., Sun M., Lu J., Wang Y., Li X., Chang F., Hao M. (2015). An evaluation of China’s new rural cooperative medical system: Achievements and inadequacies from policy goals. BMC Public Health.

[39] Hankey G.J. (2012). Nutrition and the risk of stroke. Lancet Neurol..

[40] Cohen A.K., Syme S.L. (2013). Education: A Missed Opportunity for Public Health Intervention. Am. J. Public Health.

[41] Yang Y. (2008). Social Inequalities in Happiness in the United States, 1972 to 2004: An Age-Period-Cohort Analysis. Am. Sociol. Rev..

[42] Guo F. (2010). The Study on the Early Health Enterprise in Nanjing National Government. Master’s Thesis.

[43] HO P. (1957). Development of hygiene and health work during the first five-year plan. Chin. Med. J..

[44] Zhang Y., Zhang L., Ma Z., Gao Q. (2008). Age change and phase difference of the influence of natural disaster in China in the 20th century on social economy. J. Catastrophol..

[45] Chen T. (2011). An Empirical Study on China’s Reform and Opening-up Policy Effectiveness. Stat. Res..

[46] Lin G., Fu J., Jiang D., Hu W., Dong D., Huang Y., Zhao M. (2013). Spatio-temporal variation of PM_2.5_ concentrations and their relationship with geographic and socioeconomic factors in China. Int. J. Environ. Res. Public Health.

[47] Kassebaum N.J., Lopez A.D., Murray C.J., Lozano R. (2014). A comparison of maternal mortality estimates from GBD 2013 and WHO. Lancet.

